# RadSigBench: a framework for benchmarking functional genomics signatures of cancer cell radiosensitivity

**DOI:** 10.1093/bib/bbab561

**Published:** 2022-01-22

**Authors:** John D O’Connor, Ian M Overton, Stephen J McMahon

**Affiliations:** Patrick G. Johnston Centre for Cancer Research, Queen's University Belfast, BT9 7AE, United Kingdom; Patrick G. Johnston Centre for Cancer Research, Queen's University Belfast, BT9 7AE, United Kingdom; Patrick G. Johnston Centre for Cancer Research, Queen's University Belfast, BT9 7AE, United Kingdom

**Keywords:** prediction modelling, cancer, transcriptomics, radiation therapy, radiosensitivity

## Abstract

Multiple transcriptomic predictors of tumour cell radiosensitivity (RS) have been proposed, but they have not been benchmarked against one another or to control models. To address this, we present RadSigBench, a comprehensive benchmarking framework for RS signatures. The approach compares candidate models to those developed from randomly resampled control signatures and from cellular processes integral to the radiation response. Robust evaluation of signature accuracy, both overall and for individual tissues, is performed. The NCI60 and Cancer Cell Line Encyclopaedia datasets are integrated into our workflow. Prediction of two measures of RS is assessed: survival fraction after 2 Gy and mean inactivation dose. We apply the RadSigBench framework to seven prominent published signatures of radiation sensitivity and test for equivalence to control signatures. The mean out-of-sample R^2^ for the published models on test data was very poor at 0.01 (range: −0.05 to 0.09) for Cancer Cell Line Encyclopedia and 0.00 (range: −0.19 to 0.19) in the NCI60 data. The accuracy of both published and cellular process signatures investigated was equivalent to the resampled controls, suggesting that these signatures contain limited radiation-specific information. Enhanced modelling strategies are needed for effective prediction of intrinsic RS to inform clinical treatment regimes. We make recommendations for methodological improvements, for example the inclusion of perturbation data, multiomics, advanced machine learning and mechanistic modelling. Our validation framework provides for robust performance assessment of ongoing developments in intrinsic RS prediction.

## INTRODUCTION

Radiation therapy (RT) has been used as a treatment for cancer for over 120 years [1, 2]. The vulnerability of human cells to ionising radiation was realised quickly after the discovery of X-rays and led to their use in the treatment of malignancies [3]. Today, around 50% of cancer patients receive RT at some point during their treatment, often combined with chemotherapy and surgery [4]. The development of RT has been multifaceted with improvements in dose delivery technology (for example, high-energy linear accelerators), three-dimensional imaging and treatment planning for improved precision in dose delivery to the tumour. This has enabled treatments to be delivered while reducing dose to normal tissue and associated side-effects [5]. However, despite this physical personalisation, biological personalisation in radiotherapy remains limited.

Genomic information offers great potential for optimisation of cancer treatment, partly due to rapidly decreasing sequencing costs [6]. Indeed, genetic analysis has revealed considerable diversity underpinning tumour biology [7, 8]. However, in contrast to targeted chemotherapy [9], attempts at genome-driven biological personalisation of radiotherapy have not yet translated to clinical practice. Although some transcriptome-based tumour radiosensitivity (RS) predictors have been tested in patient cohorts, they have performed poorly in independent *in vitro* validation leading to debate concerning their accuracy and specificity to radiation response [10, 11]. In this work, we review a selection of prominent RS prediction models and evaluate their performance in two different *in vitro* datasets [12, 13]. Response to radiation exposure is heterogeneous across cell lines and tumours [14–16]. It is believed that two of the key factors, which determine these responses, are intrinsic RS and proliferation status.

Intrinsic RS describes the sensitivity of individual cells to ionising radiation, typically determined through clonogenic assays, and is primarily governed by the cellular response to DNA damage. In particular, the quantity of a complex form of DNA damage, called double strand breaks (where both strands of the DNA molecule are severed in close proximity), is a key factor determining cell survival [17]. The cell cycle distribution of irradiated cells is also important, affecting both the complexity of radiation-induced damage and the repair mechanisms available to the cell, conferring radioresistance in S-phase and sensitivity in late G2 and mitosis [17]. Alterations in DNA damage repair pathways are characteristic of cancer, driving differences in intrinsic RS across tissues, cancer types and individuals [18]. Mutations in key DNA damage repair genes are well-established risk factors for cancer development and influence clinical decision making (for example BRCA mutation in breast cancer), but equivalent applications in prediction of response to radiation treatment have not reached the clinic [9–11].

Within tumours, higher cell proliferation rates generally require increased radiation dose to achieve equivalent tumour control probability [19]. Proliferation may be measured using the mitotic index and by the number of cells in the S-phase of the cell cycle [20]. Active proliferation may also change repair and cell death pathways due to changing cell cycle distributions. Thus, while many gene expression signatures have been developed using *in vitro* measurements of radiation sensitivity, proliferation may be a confounding factor when these signatures are applied for patient stratification. Notably, Venet *et al.* [21] showed that a large portion of published breast cancer signatures were no longer associated with clinical outcomes when an indicator of proliferation was accounted for.

### **Measuring tumour cell** RS

RS is commonly measured *in vitro* with the clonogenic assay, where a population of tumour cells is prepared and divided into two sets, and one is irradiated, while the other serves as a control [22]. After a defined period (usually 1–3 weeks), cells forming colonies of over 50 cells are deemed clonogenic. The surviving fraction is then calculated by the ratio of the plating efficiencies for the treated condition relative to untreated cells. Under normal exposure conditions, this is typically considered to reflect the intrinsic RS of the cell line.

Cell survival (S) after exposure to radiation dose (D) is typically characterised by a linear-quadratic model (Eq. 1) from which several parameters may be derived to characterise cellular responses

(1) \begin{equation*} S={e}^{-\alpha D-\beta{D}^2}. \end{equation*}

These include the fitting parameters from the linear quadratic model (*α*, *β*), survival fraction at 2 Gy (SF2) and the integral of the dose–response curve (mean inactivation dose; MID). These values have shown utility in predicting clinical outcomes such as survival and recurrence [23]. SF2 was reported to be independent of T and N-category, age and sex, although the same study found negligible correlation between *in vitro* measures of RS and *in vivo* loco-regional tumour control, which may reflect intratumoural heterogeneity [24, 25].

The clonogenic assay is time-consuming, making the large-scale assessment of a diverse range of cell lines difficult. However, a recently developed high-throughput RS assay based upon a luminescent readout of ATP levels was used to compare irradiated cells to controls and showed good agreement with clonogenic survival at doses greater than 2 Gy [26].

### Matched RS and genomics datasets

Data characterising the genetic background of cancer cells is publicly available from a variety of well-maintained repositories; availability of matched RS measurements is the limiting factor in the development of RS predictors. Identification of genetic determinants of RS has focused on targeted investigations in individual cell lines, together with some interrogation of resources such as the NCI60 and the Cancer Cell Line Encyclopedia (CCLE) [12, 13]. Although both of these cell-line datasets were initially developed for anti-cancer drug screening, subsequent characterisation of their response to radiation has led to them becoming the largest and most frequently employed datasets in tumour RS modelling.

The NCI60 is a panel of 60 cell lines covering lung, skin, blood, colon, central nervous system (CNS), ovary, breast, prostate and kidneys cancers [13], with detailed genomic characterisation at the DNA, RNA and protein level available, including radiation-induced changes to the transcriptome [16, 27]. Clonogenic RS data have been available for the NCI60 for a number of years [14, 16], and consequently, the NCI60 forms the basis for most of the cell line RS studies described here; however, the relatively small sample size hinders validation on held-out, independent test data. The diversity in tissue-of-origin across the NCI60 may also have contributed to previously reported poor performance for some RS signatures [10, 11].

The CCLE also provides rich functional genomics data [mutations, copy number variations (CNVs), RNA microarray and RNAseq] for a significantly larger number of cell lines [12] and has recently [15] been complemented with RS measurements (*n* = 533). Although some cancers are underrepresented, CCLE represents the largest integration of radiogenomic data to date and offers opportunity for testing of previously developed signatures with an increased sample size.

### RS gene signatures—models and validation

*In vitro* RS measures described in the previous sections are not amenable to direct clinical use given difficulties in culturing primary tumour cells [23]. Molecular studies have the advantage of both revealing mechanisms underpinning RS and acting as biomarkers for clinical applications. In this section, we discuss the development and validation of published RS gene signatures from *in vitro* RS data.

An early stage in the modelling workflow for signature development applies feature selection to determine differentially expressed genes between radiosensitive and radioresistant phenotypes. The majority of RS gene signatures to date have used microarray gene expression from the NCI60 dataset to identify differentially expressed genes according to RS (SF2) (Table 1). Many candidate RS prediction models have focused upon pan-cancer prediction (i.e. agnostic to anatomical site), which may result from necessity rather than design because each cancer site is represented by fewer than 10 samples. Significance analysis of microarrays is frequently employed with control for false positives by false discovery rate (FDR) and often a minimum fold-change requirement [11, 14, 28, 29]. Feature selection may be informed by functional annotation using Gene Set Enrichment Analysis, the Kyoto Encyclopedia of Genes and Genomes, Gene Ontology, Ingenuity pathway Analysis or Expression Analysis Systematic Explorer [16, 28, 29] to identify the overrepresentation of genes involved in functions related to radiation response and DNA damage (Table 1). Some studies fitted a predictive model [11, 14, 29], while others were limited to gene signature discovery [16, 28, 30] (Table 1). Models aimed at prediction ranged in complexity from a linear regression model of 10 ‘hub’ genes [14] to more involved machine learning approaches such as a support vector machine (SVM) with 129 genes as features [29]. Importantly, among the studies considered here, only one reported parameters sufficient for direct replication of their prediction model [14].

**Table 1:** Selected RS expression signatures trained using *in-vitro* assays of RS

Reference	Training tissue(s)	RS measure	Feature selection	Number of genes	Prediction model	Validation set(s)
Eschrich [14]	NCI60 (*n* = 48 cell lines)	Clonogenic assay (SF2)	Univariate linear regression and network analysis for feature selection	10	Linear regression on ranked expression	NCI 60 (*n* = 12 cell lines); [11, 31, 32, 34–37, 66]
Kim [28]	NCI60 (*n* = 60 cell lines)	Clonogenic assay (SF2)	Significant analysis of microarrays (FDR ≤ 0.10)	31	None reported	[67, 68]
Amundson [16]	NCI60 (+3 Leukaemia cell lines; *n* = 63)	Clonogenic assay (SF2, D_0_)	Weighted gene analysis	175	None reported	[11]
Zhang [29]	NCI60 (*n* = 60 cell lines)	Clonogenic assay (SF2)	Significance analysis of microarray (FDR < 0.01). PLS method to produce latent variables.	129	SVM	Clinical datasets: GSE4271, GSE4412, GSE17537, GSE15736, GSE17260, GSE9891
Hall [11]	Head and neck (*n* = 11) cell lines	Clonogenic assay (SF2)	Ranked product differential expression analysis (FDR < 0.05)	42	PCA	NCI60
Tewari [30]	Cervix (*n* = 8 primary samples)	IVRRA	Forward stepwise selection in genes with Pearson’s correlation coefficient with *a* > 5-fold difference	54	None reported	[11]

Previous studies have taken two broad approaches for signature validation: assessment on held out *in vitro* data, or clinical outcome prediction. The Eschrich *et al.* model was tested on 12 held out cell lines from the NCI60; the initial publication reported a negative correlation between measured and predicted SF2 values [10, 14]. The Eschrich *et al.* model was further tested independently on cervix and HNSCC cell lines and showed no statistically significant predictive power [11]. Models trained using HNSCC and cervical cell lines also showed poor ability to separate high and low SF2 values when implemented in the NCI60 dataset [11]. Despite poor performance on held out *in vitro* data, several signatures have shown potential to predict outcome in clinical studies. However, attribution of potential clinical value to the signatures’ ability to predict intrinsic RS is difficult, given biological confounding factors and the clinical differences between treatment groups [21, 29, 31–37].

### Summary and aims

RS prediction models have been developed based on small sample, heterogeneous datasets, with limited independent verification. While some published signatures can separate clinical trajectories [14, 29], in most studies, the potential confounding effects of other biological pathways governing tumour response, such as proliferation, have not been considered. Therefore, it is difficult to definitively attribute the models’ clinical capabilities to the prediction of the underlying RS. Additionally, performance differences across tissues have not been well characterised owing to small sample sizes.

Here, we present the RadSigBench framework to study the above considerations and demonstrate its use with two relatively large cancer cell line datasets. Specifically, we consider the following:

(i) Do published RS predictors outperform models generated by uniformly randomly resampling from all genes on the microarray?
(ii) How does RS prediction using published gene signatures compare to key cellular process signatures?
(iii) How do RS models perform across cells from different tissues or anatomical sites?

## METHODS

### Gene expression data for RS prediction

Microarray (Affymetrix; HG U133 Plus 2.0) data (RMA normalised) were downloaded from the CCLE (https://portals.broadinstitute.org/ccle) and cell line identifiers were used for matching with published RS measures (MID) [15]. Gene expression data from the HG U133 Plus 2.0 chip were also downloaded for NCI60 ([38]; GSE32474) and matched with published RS data (SF2) [49]. ProbeSets were annotated to gene symbols according to the Affymetrix specification for HG U133 Plus 2.0. For published models providing specific ProbeSet IDs, expression data were extracted for use in evaluation [11, 14]. For published models reporting only gene symbols, ProbeSets representing the same gene symbol were averaged using the *limma* package in R [16, 28–30]. Genes that could not be mapped were excluded; details are reported in Table S1 (see Supplementary Data available online at http://bib.oxfordjournals.org/).

### Published signature implementation

**Figure 1:**
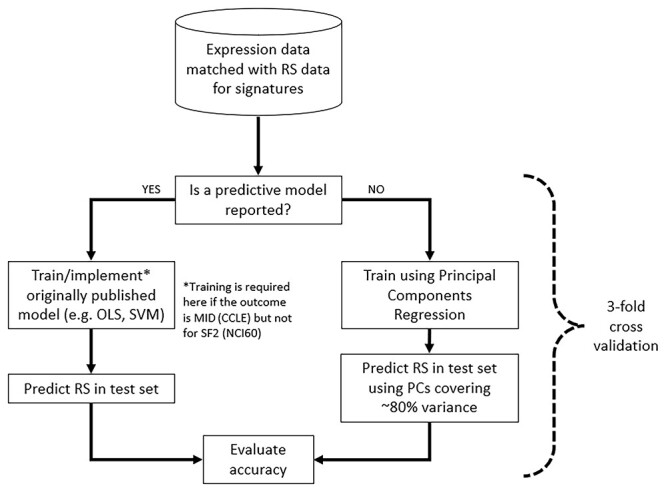
Workflow for benchmarking published signatures on CCLE and NCI60 data. Model training was dependent on whether a predictive model was reported in the paper. If not (‘NO’ route above), a principal components regression method was used to build a predictive model on the training data. This model was then applied to the held-out test set to predict the RS measure using the number of principal components that was closest to 80% of variance covered in the training set. If a predictive model was reported (‘YES’ route above) and the RS measure was the same (for example, SF2 in NCI60), the model was implemented exactly as reported (for example, Eschrich *et al.* model) or trained using the same model classes where parameters were not available (for example, Zhang *et al.* model). When the RS measure was not the same as originally published (for example, MID in CCLE), models were retrained using the original model classes (for example, linear model with ranked expression for ten hub genes in Eschrich *et al.* model).

We chose area under the dose–response curve (MID) for analysis with CCLE data, which was previously shown to have good agreement with clonogenic survival [26], while SF2 was used with NCI60 since it is the most common RS measure taken for the training of previously published models (Table 1). The list of genes for each signature is provided along with code in the GitHub repository (https://github.com/SJMcMahonLab/RadSigBench). The benchmarking workflow is outlined in Figure 1. Across all published signatures, only Eschrich *et al.* [14] provided both a model and all model parameters, enabling it to be used directly for benchmarking against SF2 in the NCI60 cell lines. Since model parameters were not reported in the Zhang *et al.* [29] model, we retrained using the original features and model classes [i.e. Partial Least Squares (PLS) followed by SVMs]. We note that the NCI60 data used here are identical to that used originally by Zhang *et al.* [29] and from the same cell lines as five out of seven of the models. The Hall *et al.*, Amundson *et al.*, Tewari *et al.* and Kim *et al.* studies [11, 16, 28, 30] either used principal component analysis (PCA) or did not report a predictive model and were retrained in RadSigBench with RS being predicted using a principal components regression (i.e. a linear regression with the principal components as explanatory variables and RS as the response variable) and taking sufficient components to cover as close as possible to 80% of the variance in the signature’s gene expression values (Figure 1). Model performance was assessed by 3-fold cross-validation and the workflow for each ‘fold’ of the cross-validation is given in Figure S1 (see Supplementary Data available online at http://bib.oxfordjournals.org/). Absolute difference between the measured and predicted values (absolute error; AE) was taken for performance evaluation and mean AE (MAE) calculated for overall model performance.

The CCLE data [12] were analysed as described above. Evaluation with CCLE data required retraining since all studies which reported predictive models were developed to predict SF2, while CCLE gave MID as the RS measure. The originally published model classes remained the same for the Eschrich *et al.* (i.e. linear regression on ranked expression) and Zhang *et al.* signatures (i.e. PLS and SVM) [14, 29].

### Cellular process signatures

In order to provide context for the error values of published signatures, the RadSigBench framework incorporates signatures representative of biological pathways that have been previously identified as important in radiation response. While not exhaustive, this approach seeks to evaluate the predictive performance of genes from key pathways against signatures trained using RS data. Genes representing cellular processes known to be involved in radiation response were obtained from the Reactome database (release #77) [39]. The selected pathways were Apoptosis (R-HSA-109581.3), Autophagy (R-HSA-9612973.2), Cell Cycle Checkpoints (R-HSA-69620.2), Mitosis (R-HSA-69278.4), Chromosome Maintenance (R-HSA-73886.2), DNA repair (R-HSA-73894.3), DNA Replication (R-HSA-69306.5) and Translation (R-HSA-72766.4). In addition, in order to explore if there is any correlation between proliferation and *in vitro* RS, we took a proliferation signature from a previous study (https://doi.org/10.1371/journal.pcbi.1002240.s002) which selected the top 1% of genes most correlated (Pearson correlation) to PCNA [21]. This proliferation signature is an established confounder in associations between the transcriptome and breast cancer outcomes [21]. The predictive ability of the cellular process signatures was assessed using the principal components regression method described in Published signature implementation section.

### Resampled signatures and intercept only models

Since previous work has shown that randomly resampled genes correlate with clinical outcome in cancer [21], we applied this approach in order to develop negative control signatures. The accuracy of the negative control signatures at different sizes (number of genes) thus provides a comparator for benchmarking potential RS signatures. Published signatures with similar accuracy to the resampled signatures are unlikely to contain significant biological information relevant to intrinsic RS response.

Control signatures were produced for both datasets by resampling from the ProbeSet-averaged data (*n* = 20 068 genes). For a given signature size, 500 different control signatures were resampled with replacement using the ‘sample’ function in R-base, and RS models were fitted using the principal components regression method. Sixteen different sizes of control signature were generated, matching the seven published signatures (i.e. 10, 19, 31, 49, 97, 129 and 168 genes) and the nine cellular process signatures (i.e. 114, 127, 131, 146, 182, 272, 289, 306 and 546 genes). The median accuracy (by MAE) signature at each size was used for performance comparison. The 95% confidence interval for MAE was calculated from the performance of the median accuracy signature on each cell line. A further negative control model was also fitted using the intercept only, which is equivalent to taking the mean value of the training data as a fixed prediction for new data.

### Statistical testing

All data analysis was performed using R 4.0.3 [40] with the limma [41] package from the Bioconductor repository [42], as well as the data.table [43], reshape2 [44], Rfast [45], caret [46] and e1071 [47] packages. Visualisations were produced using the ggplot2 [48], ggrepel [49], GGally [50] and ggpubr [51] packages. Published and cellular process signatures were compared (by MAE) to median accuracy control signatures of the same size using a two one-sided tests (TOST) procedure from the TOSTER package [52]. The smallest effect size of interest was set to ±0.1 for both SF2 and MID. *A* value of 0.1 was previously used in the development of the Eschrich *et al.* [14] model given that it is approximately equal to the reliability of the clonogenic assay. The same value was used for CCLE here because a change of 0.1 Gy in MAE is smaller than the inter-experimental variation in MID reported between different studies [53]. The TOST paired *t*-test function was used with an alpha value of 0.003 (0.05 divided by 16 to correct for multiple comparisons). Out-of-sample *R*^2^ was calculated for the published models, which may be negative in cases where the mean of the data is closer to observations than the model predictions (i.e. sum of the squared residuals is greater than total sum of squares). The correlation between accuracy and the number of genes in control signatures and MAE was assessed using linear regression. The association between sample size and MAE variation across models was also assessed using linear regression (lung cell lines were excluded from the model as the sample size was an outlier).

## RESULTS

**Figure 2:**
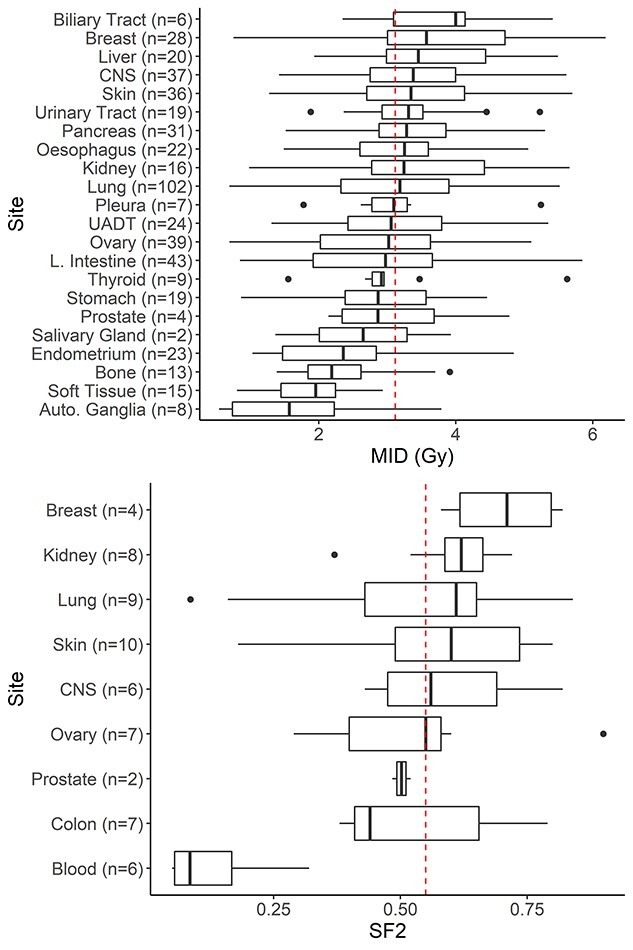
RS by anatomical location across CCLE (top) and NCI60 (bottom). The mean of all cell lines is shown as a red dashed line. A wide range is seen within and across sites for both MID (top) and SF2 (bottom). Autonomic Ganglia, soft tissue, bone, endometrium, breast and biliary tract had the largest deviation from the mean in CCLE. Breast cell lines also deviated considerably from the overall mean in the NCI60, and blood cell lines had a very large difference compared to other sites.

**Figure 3:**
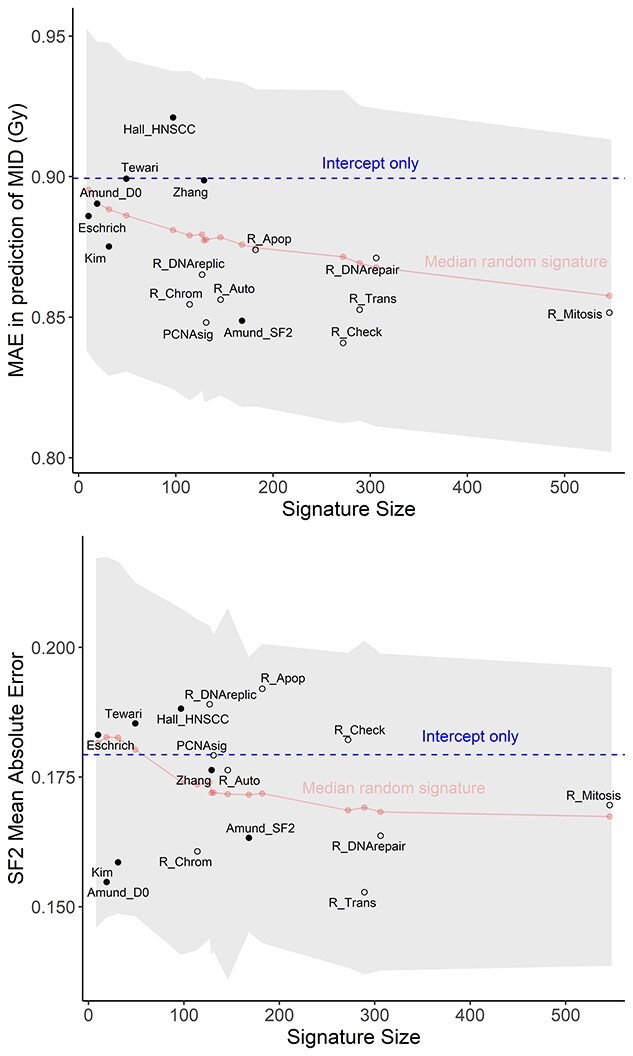
Performance of signatures predicting MID in the CCLE (top) and SF2 in NCI60 (bottom). MAE is plotted for each published model (black filled dots) and cellular process model (black unfilled dots) with size of the signature on the *x*-axis. Performance of the median accuracy resampled control signature is plotted with a red line; the grey shaded region represents 95% CI. Results from prediction models using only the intercept are shown as a blue dashed line. The performance of all models investigated was within the 95% CI for the size-matched control signature in both the NCI60 and CCLE data.

**Figure 4:**
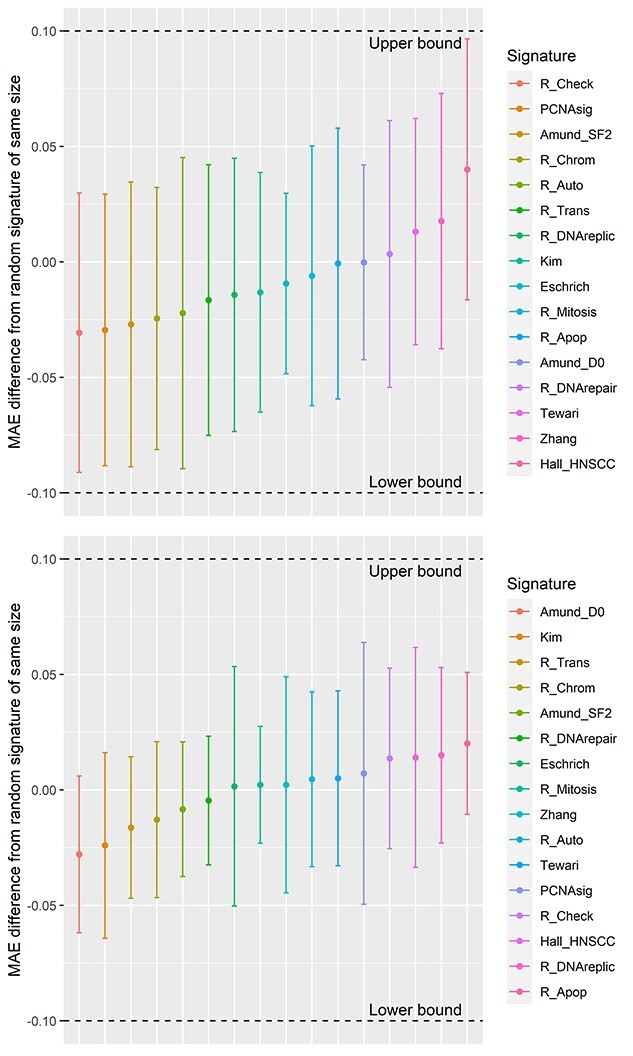
Results from equivalence testing in the CCLE (top) and NCI60 (bottom). Points represent the difference (and 90% CI) in MAE between signature predictions and the median resampled control signature of the same size. All models were equivalent to the control signature within ±0.1 for both datasets.

Measures of RS from NCI60 and CCLE showed considerable differences in mean and standard deviation between tissues (Figure 2). The performance of seven published RS signatures was compared with resampled negative control signatures, cellular process signatures and an intercept-only model (Figure 3). The control signatures showed a modest improvement in predictive performance with increasing size and MAE significantly decreased from 10 to 546 genes (*P* < 0.001). Errors for cellular process signatures were equivalent to the negative control signatures, suggesting difficulty in accurate prediction of *in vitro* RS in the NCI60 and CCLE data using genes linked to processes known to be involved in radiation response of the cell. The accuracy of all signatures fell within the 95% CI of the control signatures (Figure 3). Additionally, all published and cellular process models were equivalent to the median control signature within the bounds of −0.1 and 0.1, i.e. −10 to 10% in SF2 for NCI60 and −0.1 to 0.1 Gy for MID in CCLE (Figure 4). The mean out-of-sample R^2^ for the published models on test data was very low at 0.01 (range: −0.05 to 0.09) for CCLE and 0.00 (range: −0.19 to 0.19) in the NCI60 data (Table S2, see Supplementary Data available online at http://bib.oxfordjournals.org/).

**Figure 5:**
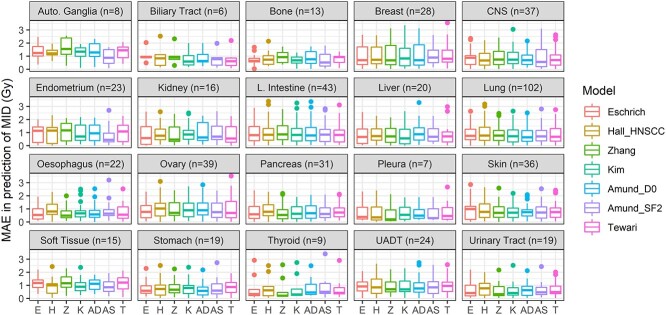
Performance of published models in predicting MID in the CCLE dataset for 20 anatomical locations. Only those with more than five samples are shown. Differences between models within tissue were small, particularly where there were a large number of cell lines, for example in lung. Differences between tissues were larger; some sites were predicted relatively poorly across almost all models (for example, autonomic ganglia), in contrast to other tissues that had relatively low error rates (for example, urinary tract and pleura).

**Figure 6:**
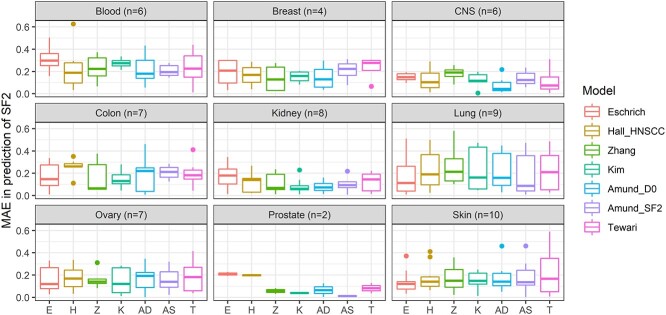
Performance of RS models across nine anatomical locations in the NCI60 dataset. The seven models evaluated are shown on the *x*-axis and MAE for SF2 prediction is shown on the *y*-axis. We found greater differences within the NCI60 anatomical sites than for CCLE (Figure 5), possibly due to smaller sample sizes for the NCI60 dataset. The models examined had generally poor performance on the blood cell lines compared with other sites.

The MAE for each published model over all tissues ranged from 0.849 to 0.921 Gy for MID in the CCLE dataset and from 0.155 to 0.188 for SF2 in the NCI60 dataset (Table 2). Averaging MAE for each tissue over all RS models in CCLE cells highlighted differences, with cells derived from the urinary tract being predicted the best (MAE = 0.66) and those from the salivary gland (MAE = 1.31) the worst on average (Table 2). The equivalent best and worst tissues in the NCI60 data were prostate (MAE = 0.08) and blood (MAE = 0.25) (Table 2). Prediction error was similar for individual tissues for all models in the CCLE (Figure 5), particularly in those with large samples (for example lung and large intestine). Sites with lower sample sizes in CCLE and NCI60 showed more variation between models (Figures 4, 5 and 6; NCI60: *P* = 0.03, CCLE: *P* < 0.01). In the CCLE, a greater difference between mean tissue RS and the mean RS across all tissues (Figure 2) was correlated with MAE in 4/7 published models (*P* < 0.05) (Figure S4, see Supplementary Data available online at http://bib.oxfordjournals.org/). The variation in RS for each tissue also correlated with MAE in 4/7 models for the CCLE (Figure S4, see Supplementary Data available online at http://bib.oxfordjournals.org/; *P* < 0.01). Although there were indications of similar trends in the NCI60 data, these were not significant, possibly due to the low sample size (Figure S5, see Supplementary Data available online at http://bib.oxfordjournals.org/).

**Table 2:** MAE for predicted RS across anatomical sites. Data are presented for the CCLE (MID) and NCI60 (SF2) databases and are sorted by the highest mean error over all models for each dataset. The three-colour scale represents error values from blue (minimum) though white (50th percentile) to yellow (maximum). Almost all of the models examined had worst performance on tissues with outlying RS values, including Autonomic Ganglia, Breast and Blood

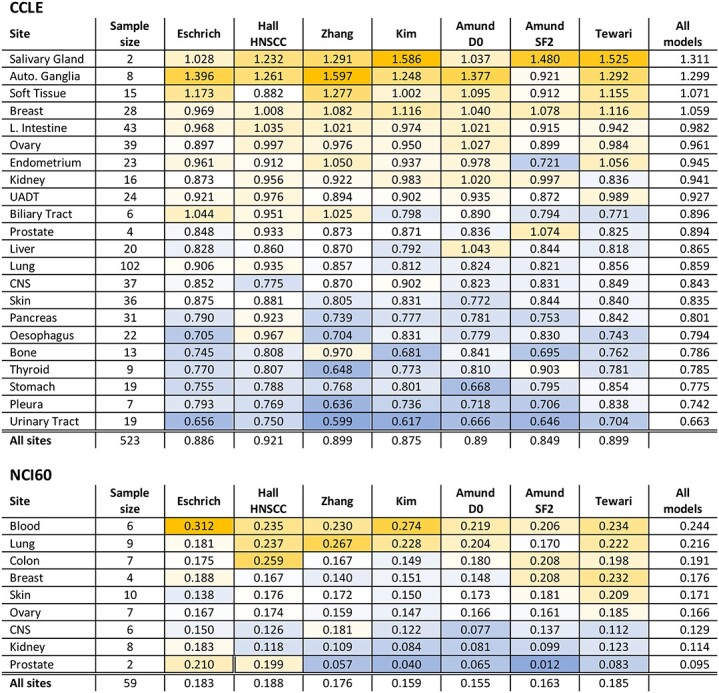

## DISCUSSION

We present the RadSigBench framework for assessing the accuracy of transcriptomic predictors of intrinsic RS in the frequently utilised CCLE and NCI60 datasets and apply it to seven published models. To help evaluate performance, signatures based on resampled genes and an intercept-only model were developed as negative controls. Cellular process signatures were also investigated to evaluate the predictive power of genes in processes believed to be associated with RS. Neither the published RS signatures nor the cellular processes evaluated had better performance than the resampled control signatures in both CCLE and NCI60 datasets. Assessment of predictions by tissue of origin showed substantial variation, suggesting potential for model improvements by incorporation of anatomical location.

### Model comparison

Correlations between predictions from the studied models were weak to moderate in both the CCLE and NCI60 datasets. Better performing models showed greater correlation with each other than models with lower performance (Figures S2 and S3, see Supplementary Data available online at http://bib.oxfordjournals.org/). Inclusion of a greater number of genes within the control signatures was associated with decreased error. The published signatures had performance equivalent to the resampled control signatures in both datasets. The Eschrich *et al.* [14] model was trained on NCI60; therefore, the poor performance on this dataset is unexpected. However, we note that the error values reported here are in line with those given in the original publication [14] and in independent validation [11, 54]. High error rates for blood cell RS prediction have been previously reported and were reiterated here, which may be due to the known high rates of apoptosis following irradiation of haematological cell lines, coupled with underrepresentation in training data [14]. Errors for Zhang *et al.* (2014) were much larger than reported in the original work (RMSE = 0.01), suggesting overfitting in the original analysis. Interestingly, this signature has previously showed utility in separating clinical cohorts by outcome, aligning with previous work demonstrating that the expression of randomly resampled genes may predict cancer patient trajectories [29]. The use of appropriate control models in our framework provides context to previously reported error values that were considered acceptable (for example the Eschrich *et al.* signature [14]), while evaluation on the test data suggests that the error rate was underestimated in some models (for example the Zhang *et al.* model [29]).

In addition to modelling methods, measurement protocols for RS and gene expression may contribute to higher error on replication. Differences between measured *in vitro* RS values can result from experimental protocol divergence (for example cell culture conditions) and inter-observer error. Although the coefficient of variation for SF2 from clonogenic assay is below 30% for A549 cells, RS values had a large range across published articles and are associated with seeding time [53]. Disparity could also arise during microarray data processing (for example the use of different background correction and normalisation techniques).

Some relatively simple steps using existing data (for example incorporation of site-to-site differences and appropriate null hypotheses) may increase the performance of signatures aiming to predict *in vitro* cell line RS. The poor performance of the models evaluated, including cell process signatures, could reflect a limitation of steady-state gene expression data for prediction of the dynamic behaviour of cell populations. Indeed, perturbation time series data [55], as well as multiomics, protein abundance and post-translational modifications have proven valuable for cancer drug sensitivity models [56] and are likely to enable improved prediction of intrinsic RS. In contrast to the relatively straightforward modelling approaches employed in the signatures evaluated here, methods such as deep learning and executable modelling with multimodal data are also expected to result in better performance [57, 58].

### *In vitro* and clinical discrepancy

Tumour response is believed to depend on both intrinsic RS and cellular proliferation. We assessed the proliferation marker PCNAsig here in order to investigate any correlation between proliferation and intrinsic RS *in vitro*. No relationship between proliferation and RS was seen, which could reflect *in vitro* experimental conditions (e.g. the clonogenic assay is typically performed when all cells are cycling and does not depend on the time taken by cells to undergo division). However, previous work shows that PCNAsig is indicative of clinical outcomes [21], suggesting that proliferation might act independently of intrinsic RS to determine tumour response. Since >50% of the transcriptome in breast cancer correlates with proliferation, it is likely that many genes identified as indicative of intrinsic RS *in vitro* might also reflect proliferation *in vivo*, which may act as a confounder in clinical validation [21]. Previous studies have provided evidence for predictive (as opposed to prognostic) capacity of radiation sensitivity signatures by showing their association in RT-treated groups and not in no RT groups (or interaction between signature and RT/noRT); although some covariates are adjusted for, clinical differences between RT/noRT patients may remain a confounder [32, 35]. Other physiological features present in clinical tumours, which are not found *in vitro*, may further impact on the relationship between intrinsic RS and tumour control—most notably hypoxia, which has been shown to impact significantly on tumour outcomes and gene expression profiles [59].

### Future directions

While some models based on clinical data have incorporated multiomics, cell line derived models have typically utilised gene expression microarray data. One study [60] took CNV and gene expression data from NCI60 to build a model predictive of SF2, reporting modest improvement on previous work with expression only [29]. Inclusion of multiomics data may improve RS prediction but will require more computational resource and added complexity in validation both on independent *in vitro* data and patient cohorts.

There is scope for considerable improvement in RS prediction for cell lines using existing data (Figure S6, see Supplementary Data available online at http://bib. oxfordjournals.org/). Although the larger sample size in CCLE did not improve predictions here relative to NCI60, increased sample diversity is likely to be beneficial for more complex models incorporating tissue differences and perturbation data. Many existing models were trained on NCI60 where differences in responses between tissues may hinder training and validation of predictive models, given the low sample sizes for many tissues. High-throughput measures of RS on CCLE cell lines could aid the development of predictive approaches [15]. Larger samples may also allow for the use of tissue site as variable for estimating RS, which might have a considerable impact upon performance; indeed, previous models have shown improved performance when some tissues were removed; for example the Eschrich *et al.* [14] model and leukaemia. Training of future signatures may also benefit from integration of genes identified across multiple platforms [28]. This approach may be extended to assays of *in vitro* RS (for example clonogenic or high throughput) and the parameters chosen to represent cell vulnerability to radiation (for example SF2, SF8 and MID). Time-lapse measurement of RS in cell culture is likely to provide more informative endpoints, as suggested for measurement of drug sensitivity [61]. In clinical validation, confidence in model specificity to RT could be improved using adjustment for proliferation and by taking resampled signatures as a comparator. Although many studies have shown signatures to be related to known pathways involved in radiation sensitivity (for example DNA repair), similarities between signatures are limited and may be dominated by differences between tissues.

Steady state transcriptome measurements used by all the signatures studied here may be augmented by incorporation of perturbation data (i.e. changes in expression after exposure to radiation, chemicals or other factors such has environmental stress). For example, Amundson *et al*. [16] make available radiation perturbation data for NCI60. Work on drug sensitivity has demonstrated the value of dynamic information in predicting cell responses [62]. Indeed, perturbations reveal dynamic response mechanisms that may help to pinpoint radiation-specific biochemical circuitry controlling key outcomes, such as cell death, and facilitate sophisticated modelling approaches [63]. For example, mechanistic models include detailed formulations of the physical, chemical and biological determinants of RS, offering a powerful framework to capture the molecular basis for cellular response to radiation [64, 65]. The framework and analysis described here provides a baseline for existing models and offers a systemic method for robust validation of future developments in the prediction of cellular RS from functional genomics data.

**Key Points**

- Few transcriptomic signatures of intrinsic cancer radiosensitivity (RS) have been independently validated.
- We review current state-of-the-art intrinsic RS prediction approaches and present a framework for robust evaluation on multiple measures, including benchmarking against control signatures and key cellular processes.
- Published RS prediction signatures were evaluated in two datasets: the NCI60 and the Cancer Cell Line Encyclopaedia. The performance of the published signatures was equivalent to control signatures generated by uniformly randomly sampling from all genes measured by the microarray.
- Prediction of cancer cell RS may be improved by inclusion of perturbation data, using larger sample sizes, multiomics, advanced analysis approaches and external validation.

## Supplementary Material

Briefings_bioinformatics_supplementary_Rev2_bbab561

## Data Availability

All data and code are accessible from: https://github.com/SJMcMahonLab/RadSigBench

## Notes

**John D. O’Connor** is a Research Fellow. His interests include mechanistic and phenomenological modelling in biology and biomedical engineering.

**Ian M. Overton** is a Senior Lecturer. His research interests include data intensive applications to connect genotype with phenotype for precision medicine.

**Stephen J. McMahon** is a Reader. His research interests are in the application of mathematical models at the organism and cell-level to predict outcomes in radiation therapy.
